# An exploration of the personal experiences of digital forensics analysts who work with child sexual abuse material on a daily basis: “you cannot unsee the darker side of life”

**DOI:** 10.3389/fpsyg.2023.1142106

**Published:** 2023-06-02

**Authors:** Clare Strickland, Juliane A. Kloess, Michael Larkin

**Affiliations:** ^1^Centre for Applied Psychology, School of Psychology, University of Birmingham, Edgbaston, United Kingdom; ^2^Department of Clinical Psychology, School of Health in Social Science, The University of Edinburgh, Edinburgh, United Kingdom; ^3^Aston Institute of Health and Neurodevelopment, School of Psychology, Aston University, Birmingham, United Kingdom

**Keywords:** digital forensics analysts, child sexual abuse material, secondary traumatic stress, burnout, psychological impact, coping

## Abstract

**Introduction:**

Digital forensics analysts are a specialist group of police officers who are involved in investigating cases of online child sexual exploitation and abuse (CSEA), and identifying and classifying child sexual abuse material (CSAM) according to levels of severity, respectively. The existing literature that has examined this phenomenon suggests that this group of police officers are at greater risk of psychological harm as a result of being exposed to CSAM, and that working with this type of material has the potential to significantly affect their mental health and wellbeing.

**Methods:**

The study presented here used Interpretative Phenomenological Analysis (IPA) to explore digital forensics analysts’ personal experiences of working in this role, and with CSAM, on a daily basis, as well as how they feel this has impacted on them, and how they manage this. Seven digital forensics analysts from a specialist unit in the UK took part in semi-structured, in-person interviews.

**Results:**

Three themes were identified, namely: (i) Once you know you cannot unknow, (ii) Constant struggle to decompress, and (iii) The ups and downs of working as a digital forensics analyst. Participants talked about the difficulty of escaping the reality of the sheer prevalence of CSEA, and that working as a digital forensics analyst ultimately takes a toll on one’s mental health and wellbeing.

**Discussion:**

As a result of undertaking this work on a daily basis, participants reported experiencing symptoms comparable to compassion fatigue, secondary traumatic stress, and burnout, and reflected about the long-term or irreversible psychological effect that working in this role may have. Findings are discussed in relation to theoretical and practical implications, as well as directions for future research.

## 1. Introduction

The sexual exploitation and abuse of children via internet technologies (hereafter online CSEA) is a global challenge (WeProtect, 2021), and has been identified by the National Crime Agency (2021) as one of the major threat areas in the UK. In 2019, the UK’s Home Office reported that police were safeguarding over 600 children each month from online CSEA (Home Office, 2019), and, since the COVID-19 pandemic, the National Crime Agency (2021) estimated that between 550,000–850,000 individuals in the UK pose a sexual risk to children. In 2020, the UK’s Internet Watch Foundation (2020) reported that the nearly 154,000 reports they received (for the assessment of illegal content) were CSAM. Furthermore, according to National Society for the Prevention of Cruelty to Children (2021), there has been a 16% increase in the number of offenses of online CSEA being reported. It is suggested that these figures are reflective of the rapid advancements in internet technologies, and the ever-increasing accessibility of the internet to users across the world, making the access to children, CSAM, and content dedicated to the sexual exploitation and abuse of children more readily available (Krause, 2009).

Since the 1990s, police forces and government agencies have seen an exponential increase in the trading of CSAM (Krause, 2009). As a result, specialist units were designed to respond to this threat, and their dedicated police officers (referred to in the UK as digital forensics analysts) investigate electronic devices seized as part of police investigations for the presence of CSAM. In the UK, this involves manually processing all “unknown” digital material (i.e., any material that is not “known” to the UK’s Child Abuse Image Database [CAID]; Home Office, 2018), and identifying material that is of an indecent nature by determining whether (a) a child is present in the material, and (b) the material is of an indecent nature. Indecency is established in accordance with the legal classification system, which is comprised of three different offense categories: (i) Category A: any material depicting penetrative sexual activity, (ii) Category B: any material depicting non-penetrative sexual activity, and (iii) Category C: any material depicting “erotic posting” (Sentencing Guidelines Council, 2013; Kloess et al., 2021).

Digital forensics analysts are therefore routinely exposed to CSAM as part of their role and daily work. Krause (2009) identified a number of factors unique to the role of digital forensics analysts, and those who investigate online CSEA more broadly, that were found to be associated with increased levels of stress: (i) repeated exposure to obscene content; (ii) pressure to cover leads, make cases, and save live victims; (iii) relative novelty of investigative approach and techniques; (iv) dependence on technology and IT support personnel; (v) need for encryption, and defensible online legend/persona; (vi) constantly changing “cyber landscape”; (vii) unusual time demands of online chat; and (viii) inter-jurisdictional cooperation and coordination. Despite this, and a recognition among those who work with digital forensics analysts that they are at a heightened risk of developing psychological difficulties, the existing literature has predominantly focused on exploring the mental health and wellbeing of “front-line” police officers (i.e., those who attend emergency situations that may often be of a traumatic nature). The fact that digital forensics analysts view scenes of a traumatic nature through a screen does not offer protection from its emotional impact; on the contrary, the material they are exposed to on a regular basis depict some of the most shocking and disturbing forms of violence and abuse that occur in modern society, namely the sexual exploitation and abuse of children (Bourke and Craun, 2014).

It is therefore suggested that frequent exposure to CSAM is likely to be both cognitively and emotionally demanding, and has the potential to lead to symptoms that are associated with stress, such as low mood, intrusive thoughts, and disturbed sleep (Hajcak and Olvet, 2008; Edelmann, 2010). According to studies that have focused on the population of digital forensics analysts, this group of police officers are considered to be at a heightened risk of developing psychological difficulties as a result of their exposure to CSAM (Chouliara et al., 2009; Wolak et al., 2009; Perez et al., 2010). For example, Edelmann (2010) found that undertaking the role of a digital forensics analyst has a negative impact on their emotional, cognitive, and interpersonal functioning, and affects their overall wellbeing. Among some of the most commonly reported symptoms are: (i) intrusive thoughts, (ii) flashbacks, (iii) insomnia, (iv) paranoia, (v) hypervigilance, (vi) becoming withdrawn, (vii) mistrusting, and (viii) viewing the world through a cynical lens (Burns et al., 2008; Krause, 2009; Perez et al., 2010; Bourke and Craun, 2014; Powell et al., 2015), all of which resemble compassion fatigue, secondary traumatic stress, and burnout. More specifically, in a sample of 443 internet crimes against children investigators, one in four reported to be experiencing high levels of compassion fatigue, secondary traumatic stress, and burnout (Brady, 2017). An important role in terms of mitigating the risk of psychological harm in staff was found to play the organization’s culture, context, and environment (Burns et al., 2008; Powell et al., 2013, 2014, 2015; Fortune et al., 2018).

In the literature, vicarious trauma/traumatization and secondary traumatic stress have been used interchangeably, however, they represent two very different phenomena. According to Baird and Kracen (2006), vicarious traumatization refers to “harmful changes that occur in professionals’ views of themselves, others, and the world, as a result of exposure to the graphic and/or traumatic material of their clients” (p. 2). It therefore specifically focuses on a cognitive phenomenon. Secondary traumatic stress, on the other hand, refers to “a set of psychological symptoms that mimic post-traumatic stress disorder (PTSD), but is acquired through exposure to persons suffering the effects of trauma” (Baird and Kracen, 2006, p. 2). The authors further argue, based on their review of the relevant literature of the two constructs, that (i) “persuasive evidence exists for personal trauma history, reasonable evidence for perceived coping style, and some evidence for supervision experiences, as important predictors of vicarious traumatization,” and that (ii) “persuasive evidence for amount of exposure to trauma material and reasonable evidence for personal trauma history are indicated as important in the development of secondary traumatic stress” (p. 1). Furthermore, according to Figley (1995), secondary traumatic stress is a disorder experienced by those supporting or helping persons suffering from PTSD, having previously used the term “compassion fatigue” to describe the symptoms of exhaustion, hypervigilance, avoidance, and numbing, which is often experienced by professionals who work with people with PTSD. Contrary to vicarious traumatization, the focus here is not specifically on a cognitive phenomenon (Baird and Kracen, 2006).

While societal and organizational awareness around the importance of mental health and wellbeing at work is improving, as well as the need to protect staff from experiencing psychological consequences as a result of the work, more needs to be done to better understand the factors that impact on the mental health and wellbeing of staff who often work with very difficult material in office spaces, and as such are often forgotten about due to not being more visible. In addition, all the well-meaning initiatives will have little effect if work culture remains unchanged. Especially in military and police organizations, mental health and wellbeing continues to be stigmatized, self-care strategies are not celebrated, and seeking support is still perceived as a weakness (Farrell et al., 2018; Papazoglou and Tuttle, 2018). Consequently, this influences how help-seeking is perceived, and affects how many police officers access professional support (Edwards and Kotera, 2021). This is especially concerning given the high prevalence of maladaptive coping strategies (e.g., alcohol, illicit substances, withdrawal, avoidance) to manage stress among police officers (Follette et al., 1994; Daly, 2005).

Much of the existing, albeit limited, literature has examined the psychological risks and impact of working with CSAM outside of the UK (predominantly in the US). Given the differences in terms of the nature and context of policing between these countries and the UK, and the roles carried out by specialist units respectively, it is important to explore the personal experiences of digital forensics analysts in the UK in order to better understand how they make sense of these experiences, what it means to undertake their role on a daily basis, how it impacts on them and their lives, both personally and professionally, and how they manage this. Furthermore, most of the existing literature has adopted a quantitative methodology. The aim of the study presented here was therefore to explore the personal experiences of digital forensics analysts who undertake this role on a daily basis, using Interpretative Phenomenological Analysis (IPA) – IPA is a qualitative methodology designed to make sense of people’s life experiences, and the meaning they attach to these experiences (Smith et al., 2022).

## 2. Method

### 2.1. Ethics statement

Full ethical approval for the study was granted by the Science, Technology, Engineering and Mathematics Ethical Review Committee at the University of Birmingham. The researchers adhered to the British Psychological Society’s Code of Human Research Ethics (British Psychological Society 2021) throughout the study. In addition, the researchers received approval from the participating police force to visit their specialist unit, and recruit digital forensics analysts from its base.

### 2.2. Design

The present study used a qualitative design, and employed the qualitative methodology IPA.

### 2.3. Interpretative phenomenological analysis (IPA)

IPA is a qualitative methodology/methodological approach that is “concerned with the detailed examination of human lived experience” (Smith et al., 2022, p. 26). It examines the meanings attached to a person’s phenomenology through the process of interpretation (i.e., hermeneutics), and adopts a double hermeneutic approach, whereby the participant attempts to make sense of their personal experiences, and the researcher subsequently makes sense of the participant making sense of their experiences (Smith and Osborn, 2003). The researcher merely interprets, and draws out meaning from, what the participant has shared, with the participant’s meaning-making being first-order, and the researcher’s being second-order (Smith, 2019). Finally, IPA is focused on the idiographic nature of the participants’ personal experiences, and involves a commitment to detail and in-depth analysis of their accounts (Pietkiewicz and Smith, 2014; Smith et al., 2022).

### 2.4. Participants

A total of seven digital forensics analysts from a specialist unit at a UK police force took part in the study. Of these, four were male, and three were female. The length of time participants had worked as a digital forensics analyst ranged between three to 15 years (*M* = 9 years). Due to the small size of the specialist unit, no further demographic information was recorded in order to protect participants’ identities and ensure confidentiality.

### 2.5. Procedure

The first author was put in touch with the head of the unit in order to arrange a visit to the specialist unit to brief potential participants about the nature of the study. Once a visit had been arranged, the first and second authors visited the specialist unit, and received any potential participants in a room separate to their office to hand them a participant information sheet, and tell them more about the research. Participants were asked to contact the first author via email to express their interest. If they agreed to take part, a mutually convenient date and time was arranged for the first author to visit the specialist unit, and undertake the in-person interview there. Participants were asked to complete an electronic consent form 24 h prior to attending the interview. The interviews were conducted in a private room away from the specialist unit’s open-plan office. Prior to the commencement of the interviews, participants were reminded of the purpose of the study, and invited to ask any questions.

### 2.6. Data collection

The interviews followed a semi-structured interview schedule (see Supplementary Material), and were audio-recorded using a Dictaphone. The seven interviews lasted between 60 to 90 min.

### 2.7. Data analysis

All seven interviews were transcribed verbatim by the first author after the two-week withdrawal period, using pseudonyms in place of participants’ names, and were subsequently analyzed using IPA. The process of analysis followed the detailed steps outlined by Smith et al. (2022):

1. **Reading and re-reading:** this step involves “immersing oneself in the original data” (Smith et al., 2022, p. 78), and helps to ensure that the participant becomes the focus of the analysis as the researcher becomes familiar with the data. Some of the first author’s initial reflections were noted down during this step.
2. **Exploratory noting:** this step allows the researcher to become increasingly more familiar with the data as they examine the semantic content and language used in the transcript. Exploratory notes were developed by documenting comments on the right-hand side of the transcripts, and included:
a. Descriptive comments focused on describing the things that matter to the participant (i.e., key objects of concern, such as relationships, processes, places, events, values, and principles);
b. Linguistic comments focused on the language (e.g., pauses, laughter, repetition, tone) and metaphors used by the participant; and
c. Conceptual comments focused on what the meaning of the things that matter to the participants is (e.g., what the relationships, processes, places, etc. are like for the participant).
3. **Constructing experiential statements:** this step involves consolidating and crystalizing the researcher’s thoughts in order to reduce the volume of detail in an analytical way while maintaining the meaning. An experiential statement should relate directly to the participant’s experiences, or to their experience of sense-making.
4. **Searching for connections across experiential statements:** this step involves the researcher mapping experiential statements in such a way that they fit together. The researcher may discard some experiential statements at this stage. A helpful process to search for connections across experiential statements is by cutting them up, and moving them around.
5. **Naming personal experiential themes (PETs) and consolidating and organizing them into a table:** this step involves labeling the clusters of experiential statements to describe their characteristics.
6. **Continuing the individual analysis of other cases:** this step involves repeating the process [i.e., steps (i)–(v)] above each further individual transcript, while “being cautious about simply reproducing ideas” (Smith et al., 2022, p. 99).
7. **Working with PETs to develop group experiential themes across cases:** this final step involves examining similarities and differences across PETs to develop group experiential themes (GETs). GETs reflect shared and unique characteristics of the experiences across cases.

## 3. Results

A range of topics and experiences important to the participants were discussed during the interviews, some of which were reoccurring and apparent across participants’ accounts. Three overarching themes were identified that reflected the impact of participants’ daily work on their lives, namely: (i) Once you know you cannot unknow, (ii) Constant struggle to decompress, and (iii) The ups and downs of working as a digital forensics analyst. Each of the themes is discussed in detail, and supported with quotes from the participants.

### 3.1. Theme 1: once you know you cannot unknow

This theme reflects the idea that working as a digital forensics analyst heightens one’s insight into the prevalence of online CSEA, and the related risks to children. A commonality across participants was their experience of hypervigilance, and being alert to any sign that may indicate the presence of CSEA. This was exacerbated by feelings that one cannot escape the reality of CSEA, and as a result impinged on participants’ home lives in some way: “it is everywhere to be honest, you cannot get away from it” (Riley; p. 20). Similarly, Alex felt that “they are everywhere” (p. 19).

Some participants described experiencing viewing CSAM as “disgusting” (Alex, p. 10), “quite disturbing” (Jess, p. 5), and “really awful, horrible” (Charlie, p. 11). It was evident from participants’ accounts that being exposed to CSAM on a daily basis changed the way they viewed the world, with most adopting a more cynical outlook:

“You just see this like this underbelly of people that you are exposed to all the time, and I know it is like a warped version of it, not everyone is like that—most are decent nice people (laugh) er—but yeah, you get a bit afraid of going to certain places because you think you are going to encounter these people… but yeah, I think you do naturally get a bit risk averse.” (Sam, p. 58)

Sam acknowledged that the daily exposure to CSAM was not only a reminder of the significant global issue that it is, but also that it warped one’s view of the world, which was very much shared by others: “I have sort of a negative view of society and people, you know, because I’m fully aware of the actual extent of the problem” (Charlie, p. 6). Participants’ cynical outlook also impacted on their ability to trust others:

“I dealt with the guy who should be the pillar of society and it makes you very distrusting of everybody, so yeah, in that respect I am cynical, I am mistrusting, erm, I am always thinking, I kind of think you know, even when people are genuinely nice to you, I’m thinking “well, what are you after?!”… especially when you are dealing with people in here who are doctors, surgeons, senior police officers, they are from all walks of life and it makes you think you can’t trust anybody really.” (Danny, p. 34)

Danny’s sense of mistrust was shared by other participants: “There is nowhere that is actually completely safe” (Jo, p. 18). This sense of mistrust was often accompanied by a feeling of constant hypervigilance. Anticipating the worst in every situation led to participants acting in a judgmental manner or prejudiced way toward others, which subsequently evoked feelings of guilt:

“I have judged somebody unfortunately, which I shouldn’t have done, erm, or should have just taken the time to you know to just get to know them more really and not kind of assume things that I shouldn’t assume. Erm, so yeah, that is definitely something that I would like to change.” (Jo, p. 41)

While other participants also spoke about feelings of guilt (e.g., “I feel guilty I think, for thinking bad things about them”; Alex, p. 26), Jo further reflected on what this feeling of guilt was about, namely having or engaging in a thought one should not have or engage in (i.e., judging others, assuming and seeing the worst in others).

Interestingly, other participants relayed how their fear of judgment by others left them feeling paralyzed, and unable to act or help in certain situations:

“I remember this one incident where there was a little toddler sort of climbing up this – obviously nothing to do with me, I was there with my own kids, but there was this toddler nearby and it was climbing up this climbing frame and I saw him fall and I went to grab him and then I stopped and allowed him to fall over, because I thought like it was this natural thing that I can’t touch somebody else’s kid, which is ridiculous isn’t it? You know, I was like “What you doing, dick, you know, that he could have hurt himself,” you know, obviously there would have been no issue at all there, but I was thinking “How is that going to be perceived? Or how is that—thinking I am touching their kid or something.” (Charlie, p.16)

Charlie’s discomfort around touching other children for fear of how this may be perceived was further shared by other participants: “One of the little girls got really scared and wanted to sit on my lap and I thought I do not want to do that” (Sam, p. 21). These quotes illustrate how participants felt unable to act in ways that would be considered “normal” in their personal lives, and clearly go against their moral compass of protecting others, which is very much an ethos of policing.

Overall, this theme illustrates the impact that working as a digital forensics analyst has on participants’ lives, and how it has shaped their perceptions and world views, primarily in terms of the world being a dangerous place, and being mistrusting of others. It appears that this was partly facilitated by the number of cases participants had worked on that involved suspects who were in a position of trust (e.g., teacher, police officer), and led to a strong sense of duty and urge to protect others from the risks of CSEA.

### 3.2. Theme 2: constant struggle to decompress

This theme reflects the idea that working as a digital forensics analyst requires a good work-life balance. However, only two participants acknowledged that such a balance was difficult to achieve, despite most participants reporting that they felt a need to distance themselves from work in some way, especially in light of concerns around the potential impact their work may have on them in the future:

“Separating work from home life it is taking its toll and I am increasingly, erm, desensitized to it, erm, and consciously trying to make an effort to care about what I am doing at work, whereas caring about what I do used to be second nature, I used to be very (cough) very keen without having to consciously make an effort to be keen.” (Jess, p. 7)

All participants described the emotional toll of grading live abuse cases, especially if this involved watching videos, making it almost impossible to watch a video and compartmentalize it as “merely” evidence. It appears that there are certain aspects to the role of a digital forensics analyst that intruded on participants’ personal lives more easily, were more difficult to switch off from, and more challenging to manage overall:

“I think it’s not always indecent images that are the worst thing, erm, it could be stories you know and sometimes words and descriptions can be a lot more real than actually looking at images, erm, so reading some chat logs you know they are always quite difficult, erm, you know, where there is some grooming going on or just some really nasty stuff, like some hard core sex story going on that is just awful.” (Charlie, p. 29)

Most participants reported that they needed and took time away from grading at some point, due to it being “physically and mentally draining” (Jo, p. 23), by intentionally selecting a case that involved another subject matter (e.g., fraud) as a reset:

“Let’s get you a fraud job, let’s get you a job of a different type to give you that break so you have got that head space, because sometimes we do have 2–3 jobs that are just positive in terms of containing that material, I mean constantly looking at it and it can it is […] physically and mentally draining.” (Jo, p. 23)

All participants highlighted ways in which they ensured that work does not intrude on their home lives. Some found living a distance away from work, and having a commute, as beneficial in terms of giving them a “window of time to decompress” (Jess, p. 6), and creating a “detached link” (Jo, p. 18), between work and home. Others identified hobbies, most of which they already had and enjoyed prior to starting work as a digital forensics analyst, including exercising, enjoying the outdoors, and socializing. All of these were described as therapeutic and helpful ways to switch off from work:

“I like exercising, I love going to the gym this that and the other. That can be a coping mechanism if you have had a bad day, it is nice to go and get your frustrations out, erm, yeah definitely, you know if I am feeling a bit sluggish or lethargic or I’ve had a day that I have found quite mentally draining.” (Danny, p. 19)

Despite efforts by participants to keep work separate from their home lives, most still reported experiences of hypervigilance, paranoia, and nightmares:

“It is quite nice to go camp somewhere and explore the surrounding area… I have usually got something in my mind like the next project that I am doing or how I am going to fix something at work, but, yeah, it just naturally pops into my head, like I’m just sat there waiting for the kids to do something, yeah, it is still probably in my head a little bit, erm, but, yeah, I’m not, it’s not too much.” (Sam, p.17)

While Sam acknowledges the idea that work continues to be in the back of one’s mind, and “naturally pops into your head,” he gives the impression that this is infrequent, manageable, and not too bad. Interestingly, some participants spoke of the importance of a stable and content home/personal life in order to be able to manage and cope with the complexity and challenging nature of the role of a digital forensics analyst:

“I think it is an important thing to have that balance and I think if the two worlds, your work life and home life, intermesh, it is not a balance it is just one big mess, so I think visually you need that yin and that yang and so I am maintained that if your personal life is kind of sorted it won’t impact what you do here, but if your personal life is, you know, is tanking, erm, then it is going to impact on your ability to cope with the material that you do and that is what I mean by a work-life balance.” (Jess, p. 11)

Here, Jess suggests that when working as a digital forensics analyst, one has reduced capacity to manage or cope with additional, other sources of stress. Overall, this theme illustrates how participants may struggle at times to decompress and escape from the work of a digital forensics analyst, despite their individual efforts to establish a work-life balance.

### 3.3. Theme 3: the ups and downs of working as a digital forensics analyst

This theme reflects the idea that working as a digital forensics analyst “definitely has a shelf life” (Jo, p. 44). Participants who had worked in this role for a number of years expressed that they felt their time was coming to an end, and described how they were seeking new opportunities to get away from being exposed to CSAM. In addition to presenting as concerned about “the strain” of doing this type of work, and the “permanent damage” it may have on them (Jess, p. 34), most participants also reported feeling “forgotten” (Jo, p. 29), “devalued” (Alex, p. 33), and “frustrated” (Danny, p. 39). The frustration participants experienced predominantly related to the lack of feedback about the outcomes of cases they had worked on, often “you never get any response back” (Charlie, p. 43), leaving them questioning whether their hard work had any impact:

“I think sometimes, I think we do that many jobs that you go I don’t want everybody to go “Oh, thank you, that was brilliant or thank you, that was a brilliant job,” because that is not why we are doing it, you know, we don’t do it for thanks or for praise, but I feel like sometimes we are a bit forgotten about and, you know, and you see all these officers who yes, have worked hard, but everyone has played a part, but it would be nice if someone said you know what DF did a good job as a collective.” (Jo, p. 29)

Despite the challenges participants described as a result of working as digital forensics analysts, and all that comes with that, they saw their job as important, and were motivated to continue working in this role to “make a difference”:

“I mean we may not be recognized, but I do feel that I know what we do here makes a difference and dangerous people do go to prison, dangerous people do end up basically marked with a marker of shame being on the sex offender register, but you know they should be. Erm, so I do believe that we do what we do here genuinely does have a positive impact.” (Jess, p. 35)

Jess’ experience of what digital forensics analysts do “making a difference,” and “having a positive impact,” was shared by other participants: “Getting some nasty people off the streets” (Danny, p. 23). It is evident from participants’ accounts that this sense of value of their role and work acts as a motivating factor, and when desired outcomes of cases (e.g., a prison sentence) are achieved, this is incredibly rewarding, and has a positive effect on job satisfaction:

“[…], but “I want to get in to this and find out who this is to make sure they are safeguarded. Let’s find this perpetrator,” so that focuses you so again you I can look past it and focus on that… once you get them identified and you find out x amount of kids have been safeguarded and somebody has been locked up, it is a nice uplifting feeling… I went to court and I was hammered in the witness box, but then when he was found guilty in 20 minutes of the jury the feeling of euphoria is like—and then he is going to prison and then it makes it all worth it, you know what I mean. That kind of overrides it for me.” (Danny, p. 36)

Danny’s description of the experience of a suspect receiving a guilty verdict as “euphoric” highlights the importance of finding out about the outcomes of cases digital forensics analysts work on, and the positive impact this can have on their sense of accomplishment. Similarly, participants reflected on the importance of being supported by their colleagues and line managers, and for them to understand and validate how difficult the job can be:

“My line manager, he is not the type of person that would expect you to sit there and watch that all day, he understands people and the good thing is he doesn’t mind if you sit down and talk to someone for like a few minutes or go have a tea or whatever, he doesn’t care because he understands. Erm, and it and it is good because you can see like people naturally just start talking for a bit, then go back to their work, so you can see that people do have breaks. Erm, because for someone to sit there all day and say they are not affected by that is that’s lies.” (Riley, p. 23)

Riley’s experience of feeling supported by his line manager was shared by other participants: “Our immediate supervisor, he’s as good as gold, you cannot knock him, he is very supportive” (Danny, p. 17). However, there is a sense that despite having a very good working relationship with one’s line manager or supervisor, participants did not necessarily seek support from them when they were struggling. More specifically, some participants expressed fear of being redeployed if they disclosed that they were experiencing psychological difficulties, either more generally or as a result of being exposed to CSAM: Staff are “frightened to voice them in case they are removed from their post… it will be, “you are a potential liability, off you go” and that feeling runs quite prevalent through a lot of the staff here” (Danny, p. 17). This appeared to act as a significant barrier to seeking support from the organization, with participants reporting that they preferred to do so externally, as and when they needed.

One aspect that appeared to be at odds with looking after the mental health and wellbeing of staff was participants’ experience of the specialist unit being run like a “business” (Jo, p. 26). This was further reflected in Danny’s description of the organization’s approach being one of a “sausage factory mentality of get the job out of the door and get on with the backlog” (p. 38), with Jo explaining how they had to “move on to the next one, you do not have time to sit and Polish off something” (p. 31). The focus on targets and quarterly figures presumably diverts the organization’s attention away from thinking about effective ways of mitigating the psychological impact the work has on digital forensics analysts, which does little in terms of addressing the organizational culture and stigma around mental health and wellbeing:

“I still think there is stigma around mental health, you know, and things are getting better and I have always sort of because I have been in this world for so long I have always said, I have always been quite pro really and aware of this and I would always mention it at meetings and it would always get overlooked and you know it used to really annoy me and I remember reading this one article going back a few years now and it was called mental sunburn… erm, and it was a really interesting article and I sent it to the management here and I said “Oh this is interesting” and they couldn’t even be bothered to read it. Like I mentioned it in a meeting afterwards saying “Oh what did you think of about that?” and they were like “Oh yeah” and you just think “You really don’t care that much.” You know they talk about support, but, yeah, it is really devaluing isn’t it, it’s really really frustrating and annoying.” (Charlie, p. 42)

Here, Charlie describes how he experienced the lack of focus on and engagement with the topic of mental health and wellbeing in the organization as devaluing, frustrating, and annoying. This, in addition to the perceived lack of understanding, will undoubtedly further impact on participants’ readiness and willingness to seek support. Overall, this theme illustrates how the role of a digital forensics analyst ultimately has a “shelf life,” despite the experience of achieving positive outcomes of cases somewhat counteracting this, and acting as a reminder that their job is vital in bringing offenders to justice. Furthermore, it has highlighted the importance of having supportive superiors, and for the organization to recognize and understand the potential psychological impact this work can have on digital forensics analysts, as well as prioritize staff mental health and wellbeing. The perceived lack of trust, and ongoing stigma, clearly act as barriers, and appear to prevent staff from seeking support.

## 4. Discussion

The study presented here aimed to shed light on (i) the personal experiences of a group of digital forensics analysts recruited from a specialist unit at a police force in the UK, and (ii) how they make sense of these experiences, in order to better understand what it means to undertake this job on a daily basis. This study is the first, to our knowledge, to recruit a sample of digital forensics analysts from a police force in the UK, and use IPA as the methodology of choice. Most participants talked about the difficulty of escaping the reality of the prevalence of CSEA, and the fact that offenders represented individuals from all walks of life. This not only impacted on participants’ general perceptions and world views, but also led to them feeling suspicious and paranoid of those in positions of trust in their personal lives (e.g., toward teachers, police officers). While most participants described moments of feeling a sense of value, reward, and accomplishment, especially in the context of achieving a positive outcome for a case they had worked on, which also acted as a motivating factor, all reported that working as a digital forensics analyst ultimately takes a toll on one’s mental health and wellbeing. Most of the symptoms participants described related to hypervigilance, paranoia, safety behaviors, intrusive thoughts, disturbed sleep, nightmares, mistrust, and a cynical outlook on life, all of which are characteristic of compassion fatigue, secondary traumatic stress, and burnout. Our findings are therefore in line with previous studies suggesting that digital forensics analysts may be at an increased risk of experiencing psychological harm, including compassion fatigue, secondary traumatic stress, and burnout, as a result of working with CSAM (Burns et al., 2008; Wolak et al., 2009; Perez et al., 2010; Bourke and Craun, 2014; Powell et al., 2015; Brady, 2017). Interestingly, participants themselves presented as wondering and concerned about the long-term or irreversible psychological effect of working as a digital forensics analyst.

Those participants who had been in the job for less than 5 years appeared to experience higher levels of enjoyment and job satisfaction, while participants who had been in the job for a number of years all described experiencing some, if not all, of the above-mentioned symptoms. It could be argued that the cumulative effect of continuous exposure to CSAM was therefore more visible in participants who had been working as digital forensics analysts over a longer period of time (i.e., more than 5 years). In light of these findings, it is perhaps surprising that participants felt that they did not have access to adequate, good-quality support or clinical supervision. This perceived lack of available support by the organization further impacted on participants’ sense of feeling valued and understood, and ultimately acted as a barrier to help-seeking. More specifically, participants reported that they did not feel comfortable to disclose to colleagues or superiors when they were struggling, and that they would only seek help externally (i.e., not from within the organization). A common feature of trauma is a sense of betrayal or belief in the individual that those whose role was to protect them were unavailable to them. As such, where participants felt that the organization is not providing an appropriate level of support to them, and is therefore in breach of the psychological contract (i.e., what a worker expects from their employer in return for their labor), a sense of organizational betrayal can emerge that increases the risk of psychological harm (Carter, 2021).

While this will partly be as a result of the culture within the organization, and the persistent stigma around mental health and wellbeing, it also appeared to be related to the lack of alternatives that are available in terms of accommodating someone who may be struggling. Participants expressed fear and concerns around (i) what would happen, (ii) being redeployed, and (iii) being perceived as a “liability,” if they disclosed that they were struggling, which is suggestive of a workplace environment that lacks in psychological safety (i.e., a psychologically safe workplace is one where individuals feel able to take interpersonal risks, such as sharing vulnerability or concerns about workplace practices with management”; Edmondson, 2018). This lack of psychological safety is especially concerning given that at least some of the participants had previously worked as frontline police officers, where they will have likely been exposed to emergency situations of a traumatic nature, and are now continuing to be exposed to distressing material in their role as digital forensics analysts.

Participants further talked about the importance of hobbies and interests outside of work, as well as strategies that helped them manage their work, including (i) talking to others, (ii) taking regular breaks, (iii) alternating tasks, where possible, and (iv) detaching oneself by viewing CSAM as “just evidence” (Burns et al., 2008; Holt and Blevins, 2011; Powell et al., 2013, 2014; Ahern et al., 2016). While these strategies may be helpful in the short term, it is questionable whether they are effective in the long term. For example, Ehlers and Clark (2000) Cognitive Behavioral Model of PTSD suggests that safety strategies, be it cognitive (e.g., thought suppression) or behavioral (e.g., avoidance, keeping busy), may become problematic, and prevent the individual from being able to process the trauma memories, and manage their symptoms effectively and safely, respectively.

It is important to note that experiencing the symptoms participants reported in our study is a normal response from the nervous system as a result of being exposed to atypical, disturbing, and potentially traumatic material, such as CSAM (Krause, 2009). From an evolutionary point of view, material that involves children being harmed likely causes an even greater response from the nervous system, given that species’ protection of their offspring directly relates to and ensures their survival (Azhari et al., 2018). If symptoms of compassion fatigue, secondary traumatic stress, and burnout persist over time, and worsen in frequency and severity, there is a likelihood that this can result in long-term psychological harm, and behavioral changes, that resemble PTSD (Morrissette, 2004). Consequentially, this will impact on digital forensics analysts’ quality of life and ability to fulfill their role (Krause, 2009). According to Ehlers and Clark (2000) Cognitive Behavioral Model of PTSD, the way a traumatic event is processed may significantly impact on the individual, both cognitively and behaviorally, through (i) negative appraisals, (ii) the feeling that “the world is not a safe place,” (iii) flashbacks, (iv) intrusive thoughts, (v) insomnia, and (vi) withdrawal. In line with the current literature on trauma, experiences of trauma push an individual out of their “window of tolerance” (Siegel, 1999), which results in the nervous system becoming overstimulated, and the individual experiencing a heightened state of hypervigilance. The more frequently an individual experiences trauma responses, the smaller their “window of tolerance” becomes, and the more continuously their threat system is activated (Ogden et al., 2006). This is important to bear in mind when thinking about adequate, good-quality support for digital forensics analysts.

Overall, our findings highlight the important role workplace culture plays in creating an environment that has the potential to reduce adverse responses to and psychological impact of being exposed to CSAM. The phrase “culture eats strategy for breakfast,” coined by the management consultant Peter Drucker in the 1960s, further emphasizes the need to embed efforts and strategies to support digital forensics analysts’ mental health and wellbeing in a culture that recognizes the psychological impact of working with CSAM, and seeks to reduce implicit and explicit barriers to engaging in help-seeking and self-care activities.

While the present study offers unique insights into the personal experiences of a group of digital forensics analysts from a specialist unit at a police force in the UK, all participants were recruited from the same specialist unit. Future research would benefit from a wider recruitment strategy by recruiting digital forensics analysts from specialist units across the UK, especially in order to capture variations in terms of organizational culture. Furthermore, all interviews took place at participants’ workplace. On reflection, this may have subconsciously impacted on participants feeling comfortable to talk openly and freely. In addition, they may have felt self-conscious about leaving the office to attend the interview in case their colleagues derived their participation in the research from their absence. While interviews were originally planned to take place at the University of Birmingham. This was no longer possible as a result of the COVID-19 pandemic. It would also be of interest to explore the perspectives of partners of digital forensics analysts, and how they experience the psychological impact of the work on their partners.

Our study supports the existing literature and its findings that the group of digital forensics analysts presents with vulnerabilities to experiencing psychological harm as a result of their daily work, warranting a renewed focus on developing ways to better support their mental health and wellbeing, and providing adequate and meaningful trauma-focused support to them. In particular, specialist units that employ digital forensics analysts have to accept that there is a need for a change in culture to one that prioritizes digital forensics analysts’ mental health and wellbeing, and creates an environment where behaviors such as help-seeking and self-care are promoted. In line with trauma-informed care and practice (Office for Health Improvement & Disparities, 2022), this should include taking steps to becoming a more psychologically-minded unit, and for digital forensics analysts and their supervisors/managers to develop their awareness and understanding of how the work they undertake can impact on their mental health and wellbeing, and what type of work practices mitigate and reduce the likelihood of psychological harm. This may be further facilitated by providing an accessible and appropriate level of mandatory clinical/psychological support to digital forensics analysts, as is common and considered best practice in most workplaces that deal with potentially distressing material (HCPC, 2019). While it is appreciated that policing has suffered from significant cuts in funding and resources, the benefits of effective clinical supervision have been widely reported (HCPC, 2019). It is especially disappointing that our study found little evidence of these recommendations in practice, given that they represent practical guidelines put forward by College of Policing (2018). Finally, participants’ accounts have highlighted how the lack of feedback on outcomes of cases impacts on their levels of motivation, and sense of recognition and value. Naturally, inter-agency working is challenging at the best of times, however, specialist units are encouraged to liaise with other relevant agencies to highlight the importance of improving feedback in an attempt to decrease digital forensics analysts’ compassion fatigue, and increase their compassion satisfaction, respectively.

## Data availability statement

The datasets presented in this article are not readily available because the interviews represent personal perspectives and experiences of participants. It is therefore not deemed appropriate for the data to be made publicly available. Requests to access the datasets should be directed to the second author, JK, juliane.kloess@ed.ac.uk.

## Ethics statement

The studies involving human participants were reviewed and approved by the Science, Technology, Engineering and Mathematics Ethical Review Committee at the University of Birmingham. The patients/participants provided their written informed consent to participate in this study.

## Author contributions

JK and ML conceived and designed the study. CS collected the data, with support from JK in terms of facilitating access to the UK police force, and the specialist unit respectively. CS analyzed the data. CS and ML agreed on the final themes. CS completed the majority of the write-up, with contributions from ML. JK made substantial revisions and prepared the manuscript for submission to Frontiers in Psychology. All authors contributed to the article and approved the submitted version.

## Conflict of interest

The authors declare that the research was conducted in the absence of any commercial or financial relationships that could be construed as a potential conflict of interest.

## Publisher’s note

All claims expressed in this article are solely those of the authors and do not necessarily represent those of their affiliated organizations, or those of the publisher, the editors and the reviewers. Any product that may be evaluated in this article, or claim that may be made by its manufacturer, is not guaranteed or endorsed by the publisher.
