# Supplementary material for: An exploration of the personal experiences of digital forensics analysts who work with child sexual abuse material on a daily basis: “you cannot unsee the darker side of life”
Source: Front Psychol. 2023 Jun 2;14:1142106. doi: 10.3389/fpsyg.2023.1142106 (PMC10292749; doi:10.3389/fpsyg.2023.1142106)
Supplement: Supplementary file 1 [file Table_1.DOCX]

**Interview Schedule/Arc**

1. While we talk, I would like you to make a diagram, map or picture, which helps me to visualise the different elements of your life. Could you start by representing yourself on the paper in whatever way you like?

*Possible prompts: You can use words, symbols or images, whatever you prefer, to represent yourself on the map.*

*Possible prompts: Where do you fit in alongside your work? What is your relationship with your work? How do you experience it? What does it mean to you?*

1. Can you tell me a bit about what you have done there and why?

*Possible prompts: Interviewer to enquire into participant’s choices by making simple observation statements (e.g., ‘you chose X colour) (these are typically enough to elicit more detail from participants).*

1. Again using the supplies we have got available, please could you map your relationships with important others/those relationships that are most important to you. While you do this, it would be useful if you could think about how these relationships may play a supporting role in you being able to carry out this work on a daily basis and how you cope with it (perhaps even better than others?).

*Possible prompts: Interviewer to state what they see, prompt and enquire with a curious mind. Prompts will focus on understanding the quality and nature of each of the relationships, how it is sustained and impacted by the participant’s work, with the aim for the participant to describe their relationships in rich detail. The interviewer may subsequently enquire about other people/relationships in the participant’s life.*

1. In the context of the relationships you have mapped here, can you tell me a bit about how you ‘manage’ your work identity (e.g., not being able to discuss your work in detail) in your relationships with these people?

*Possible prompts: What is this like for you? What does this mean to/for you?*

1. So now, trying to take a step back and looking at your whole map/the whole map you have drawn, what do you think/feel when you look at it?

*Possible prompts: Is there anything in particular you are thinking or feeling? (The interviewer may also raise any specific themes that seemed important during the interview, which would benefit from further detail and context.*

1. In an ideal world, would this map look like this or is there anything you would change? *(The ‘ideal future’ question allows participants to explore how they would like their relational lives to be – their expectations, beliefs, fantasies and hopes, however, it may also reveal new information about how things are in the present.)*
2. Can you tell me a bit more about how you think/feel you may have been affected by your work over time? Have you noticed a change in yourself and/or the way your see/experience your work since you started working in this area?

*Possible prompts: What is this like for you? What does this mean to/for you? Do you remember finding it more difficult in the beginning than now? What do you think may have made this process easier for you over time?*
